# Planning for an offshore oiled wildlife response: case studies from New Zealand and Brazil

**DOI:** 10.1007/s11356-023-26440-4

**Published:** 2023-03-22

**Authors:** B. L. Chilvers, V. Ruoppolo

**Affiliations:** 1 Wildbase, School of Veterinary Science, Massey University, Private Bag, 11222 Palmerston North, New Zealand; 2 Aiuká, Av. do Trabalhador 1799, 11.725-000, Praia Grande, SP Brazil

**Keywords:** New Zealand, Brazil, Offshore oil spills, Pollution response, Planning, Wildlife

## Abstract

When an offshore oil spill occurs, it is often assumed that there will be no wildlife impacted or that an oiled wildlife response could not be undertaken. In most cases, one or both assumptions are wrong. With increasing offshore fishing, petroleum exploration, and shipping routes, the risk of accidents and spills offshore has increased. This review outlines the important considerations for offshore oiled wildlife response and explores two case studies on offshore oiled wildlife response planning based on offshore drilling or active platforms in New Zealand and Brazil. There are significant challenges for running a response in offshore environments; however, with planning, including preparation of specialized response plans, equipment, and readiness of skilled personnel, an offshore oiled wildlife response can lead to greater survival and protection for wildlife and the environment.

## Introduction

The impact of oil spills on wildlife and the environment is one of the most visible and extensively analyzed effects of oil pollution (Furness and Camphuysen 1997; Henkel and Ziccardi 2018). The most recent globally well-known offshore oil spill is the 2010 Gulf of Mexico, Deepwater Horizon spill 66 km off Louisiana, USA, with 7,800,000 m^3^ of oil estimated to be spilt and known extensive impacts to wildlife and the environment (Wallace et al. 2017). Oil impacts wildlife both through direct contact with skin, fur or feathered, and indirectly through ingestion, inhalation, and absorption (Fry and Lowenstine 1985; Burger and Fry 1993; Clark 2001). All oil types disrupt the waterproofing and thermoregulatory abilities of feathers and fur of wildlife. Feathers and furred wildlife that become oiled can lose the ability to swim, float, dive, or fly when in contact with oil, as well as the ability to control body temperature. These impacts combined can lead to hypothermia, drowning, and most likely death (Heubeck et al. 2003; Helm et al. 2015). Similarly, preening or grooming feathers and fur can lead to ingestion and inhaling the oil on their bodies, causing disruptions to hormones and reproductive ability (Altamirano 1983; Eppley and Rubega 1990; Mearns et al. 1999). The main direct routes of exposure for wildlife without feathers or fur, such as whales and dolphins, fish, amphibians, and reptiles, are ingestion, inhalation, and absorption through their skin. Without the help of humans, these impacts will result in the death of most wildlife affected by oil (Helm et al. 2015).

A fundamental principle of wildlife response is to try and prevent wildlife from getting oiled, preferably through the prevention of an oil spill occurring. If an oil spill occurs, minimizing wildlife interactions with any oil by either restricting and removing the oil before it impacts wildlife or preventing or scaring unoiled wildlife away from the area that oil will affect. If wildlife becomes oiled, the best outcome for wildlife is usually if they are captured, cleaned, rehabilitated, and released as healthy, reproductive animals back into a cleaned environment.

There are ten phases to a successful oiled wildlife response (International Petroleum Industry Environmental Conservation Association (IPIECA) 2014; Table 1). For all phases, the first and most important consideration is human health and safety; therefore, in all phases, appropriate personal protective equipment (PPE) and safety protocols should always be put in place. The first phase, planning and preparedness, should always be undertaken before an oil spill occurs. Planning and preparedness should identify and document an analysis of areas at risk, species composition and vulnerability, and potential response options and constraints, which should be assessed with authorities and companies to maximize prevention and response preparedness (Chilvers and Battley 2019; Fraser et al. 2022). Undertaking this before an event ensures health and safety concerns are incorporated, identifies what resources are available in each area, and what alternatives may need to be considered. International best practice recommends a minimum of planning, reconnaissance/monitoring, removal of dead oiled wildlife, and euthanasia of oiled wildlife if animals are suffering and cannot be rehabilitated (IPIECA 2017).

**Table 1:** The phases of an oiled wildlife response (IPIECA 2014)

Phase	Description and goals
Planning and preparedness	Determine health and safety concerns Identification of species at risk Identify and document response options Identify resource needs and availability (people, equipment, and facilities) based on possible response options
Reconnaissance/pre-emptive responses	Initial impact assessment Field assessment/reconnaissance Deterrence and/or hazing of unoiled wildlife Pre-emptive capture or relocation of unoiled wildlife
Wildlife recovery	The capture of oil impacted wildlife Transport of oiled wildlife to field stabilization Collection of dead oiled wildlife
Field stabilization	Thermoregulatory support and fluid therapy Removal of gross contaminants Address life-threatening conditions Possible triage and euthanasia^1^ evaluations based on established criteria and best practices in cooperation with key stakeholders
Transportation	Transport of oiled animals from field stabilization to a rehabilitation facility Transportation of dead oiled wildlife to a Rehabilitation facility for identification and necropsy
Intake/triage	Triage based on species’ threat classification, individual’s survivability, resource availability, cultural, legal, and animal welfare considerations Medical examination and treatment and medical records started Critical care concerns addressed Euthanasia^1^ evaluations based on established criteria and best practices and in cooperation with key stakeholders Wildlife given individual, temporary identification
Stabilization	Fluid, nutritional and medical stabilization of impacted animals 48–72-h period Prepare animals for cleaning process
Cleaning/drying	Removal of all oil/contaminants from wildlife by washing Removal of the cleaning agent by rinsing Drying, cleaned and rinsed animal
Conditioning	Restoring waterproofing and physical health (days to weeks)
Release	Permanent marking/banding of individual animals Release of cleaned, waterproof animals into a clean environment Post-release monitoring

^1^Before euthanasia is undertaken in any country, its legal and cultural acceptability needs to be established, including the training and qualifications needed for people to undertake it

The next four phases are field operations: reconnaissance and pre-emptive responses, wildlife recovery (both alive and dead oiled wildlife), field stabilization, and transport. The remaining phases of wildlife response efforts (intake/triage, stabilization, clean/dry, conditioning, and release) are accomplished in a wildlife rehabilitation facility. Planning and preparedness should be undertaken before an oil spill occurs; however, all latter phases should be undertaken as quickly as possible after a spill to prevent, reduce, and minimize oil impacts on wildlife. The outcomes of oiled wildlife response efforts will vary depending on many factors including: oil type, the extent and duration of release of oil, distance from shore of oil release, the habitat the contaminant was released into, weather conditions, species involved, number of wildlife affected, the timing of the response effort, and the time of year (Henkel and Ziccardi 2018). It is highly recommended that rehabilitated and release wildlife should be appropriately marked or have tracking tags attached where possible so their survival and return to the wide can be monitored.

Today, more than 1000 offshore rigs are operating around the world, with notable offshore fields including the North Sea; the Gulf of Mexico; Ventura Basin, California; the Caspian Sea; the Campos and Santos Basins, Brazil; Newfoundland and Nova Scotia, Canada; the Taranaki Basin in New Zealand; and many West African, Southeast Asian, Arabian, and Russian regions. There are also many new, developing areas for offshore oil exploration, such as regions of the opening Artic Ocean Northwest Passage and Southern Hemisphere including the Southern Basin in New Zealand. Of a total of 1702 spills reported between 1970 and 2018 from publicly available sources, 6% were from offshore sources (wells and platforms) with extremely large average spill sizes of 320,000 tons. Of those offshore spills, 19% reported wildlife affected (Chilvers et al. 2020).

When an offshore oil spill occurs, because oil or oiled wildlife does not wash up on shore, it is not visible to the public and media and is therefore often assumed that there is not going to be wildlife impacted or that an oiled wildlife response could not be undertaken (IPIECA 2004; Chilvers et al. 2020). In most cases, one or both assumptions are wrong. For offshore oil installations, one major factor influencing this is that in general, wildlife aggregates around offshore structures. Seabirds are known to congregate around oil drilling platforms and rigs due to night lighting, flaring, roosting refuge sites, food availability, and concentrations (both human food and waste, and food produced) because platforms create artificial reefs and augment levels of marine flora and fauna (Wiese et al. 2001). Similarly, marine mammals and turtles are also likely to be attracted to the protection and food concentrations offshore structures, rigs, and platforms offer (e.g. Lohoefener et al. 1990, Delefosse et al. 2018).

The IPIECA oiled wildlife guidelines (IPIECA 2014) do not offer options for offshore responses or special condition responses such as when resources are restricted, or when response times are likely to be extended due to distance offshore. Outlined below are the main constraints for offshore oiled wildlife response. For an offshore oiled wildlife response, a minimum response of planning, reconnaissance, and monitoring, and collection of dead oiled wildlife if washed ashore or can be collected off the water, should always be undertaken. Hazing or deterrence of wildlife to prevent wildlife from being oiled and capture and rehabilitation of wildlife should always be undertaken where possible (IPIECA 2017). The main constraints are as follows:

1. Distance from shore—a) wildlife are less likely to come ashore due to the distance making them harder to find and collect or capture and b) limited infrastructure available for an oiled wildlife response and the necessity of an on-water response for oiled wildlife operations at the site of the spill. The distance from shore may mean delayed time to response, limited resource availability in situ, additional health and safety risks, greater consideration of environmental/sea conditions, and due to travel distance, a longer time for resupplies, additional transport costs, and increased risks when using boats and helicopters in offshore environments.
2. Distance oil may spread—given offshore spills usually have limited infrastructure or land nearby, the spread of oil released is likely to be significantly wider than when oil releases wash ashore soon after release. The strength and direction of tides, currents, and winds will have a greater influence on the oils’ dispersal direction and distance (Venkatesh 1998).
3. Capacity/resource availability—resources, including availability of trained personnel and infrastructure available at the site of the spill will differ depending on the stage of offshore establishment, i.e., a drilling site verse an established drilling rig. In all cases, most resources and personnel will likely need to be brought in for a response; however, for a more established site, some resources and trained personnel may be already there. The availability of infrastructure may determine if an oiled wildlife facility could be established at a nearby location (rig or support vessel), as alternatively, animals would need to be transported long distances to onshore facilities for care (advantages and disadvantages of in situ, on a vessel or on-land facilities, are outlined in Chilvers 2021).
4. Lack of freshwater—one of the major restricted resources for any oiled wildlife response is fresh water and this would likely be an extreme limitation in an offshore environment surrounded only by seawater. Alternative methods for processes such as decontaminating wildlife by washing with seawater could be used for some activities; however, the need for freshwater for both responders and wildlife would still be extensive (Finlayson et al. 2018).
5. Weather and the environment—offshore implies remote areas, often known for strong winds, viable temperatures, ever-changing wave heights, tidal movement, and influences from currents. These environmental conditions will bring added challenges for an oiled wildlife response not only from a human health and safety point of view but also with animals suffering hypothermia, starvation, and drowning.

As indicated above, the first step (and critical before oil is spilled) for any response is planning, through the identification of species at risk, and documenting response options including legal and cultural considerations, remoteness and climate, and ensuring health and safety concerns and resource availability (IPIECA 2014). The planning and response process for offshore oiled wildlife response needs to include trained people with experience and expertise in offshore marine environments, oiled wildlife response, with specific species, and/or with specific equipment.

Here, we review the oiled wildlife response planning undertaken for two offshore locations and evaluate response options given for each area including special considerations taken given the constraints of offshore response. ‘Wildlife’, in the context of this paper and oil spill response, is defined as air-breathing vertebrates, i.e. birds, mammals, and reptiles. The two locations investigated in this paper are as follows: 1) new offshore drilling sites in the Southern Basin off the South Island of New Zealand and 2) established oil wells offshore in the Santos and Campos Basins, Brazil. These two locations were chosen because both countries have offshore petroleum operations and both countries have full-time oiled wildlife response teams responsible either through government contracts or industry contracts to plan and respond to oiled wildlife if incidents occur. This manuscript describes the two locations, the potential wildlife that could be impacted, and outlines the response options for each offshore oiled wildlife response. Based on these two case studies, special considerations and procedures for offshore oiled wildlife response are outlined. Information includes factors to consider for offshore at-sea wildlife capture, particularly for Procellariiformes species (tubenose birds such as Albatross, petrels, and shearwaters). We finish with recommendations for better global preparedness and planning for offshore oiled wildlife response.

## Planning for offshore oiled wildlife response New Zealand’s Southern Basin

**Fig. 1:**
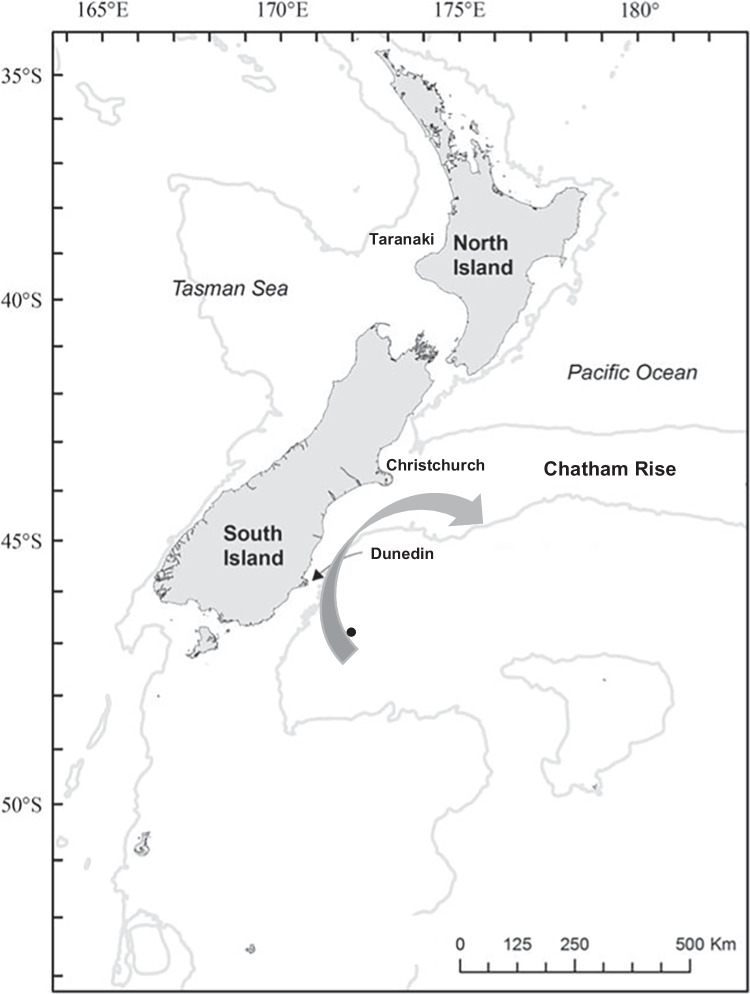
Location of Tawhaki-1 drilling site ●offshore South Island, New Zealand and projected oil movement if an oil spill occurred indicated by arrow

There is currently one establish offshore oil extraction area in New Zealand on the west coast of the North Island (Taranaki, Fig. 1). Additionally, drilling permits have been issued for an area referred to as the Southern Basin southeast coast of the South Island. Tawhaki-1 well drilling site is approximately 150 km off the southeast coast of New Zealand’s South Island (Fig. 1). This drill site was drilled during the austral summer of 2019/2020, with an oiled wildlife response plan developed prior to drilling. The potential area oil was predicted to spread if a drilling accident occurred was predominantly at sea, with oil predicted to spread in a clockwise circular motion from the drilling site, north–west up more than 500 km of the southeast coast of the South Island and then east across the Chatham Rise, with oil not predicted to reach land due to currents or tides (Fig. 1, MetOcean 2019). In New Zealand, marine oil spill response is the statutory authority responsibility of the New Zealand government department Maritime New Zealand, with marine spill response legislation addressed under the New Zealand Maritime Transport Act 1994. The management of New Zealand’s oiled wildlife during an incident is also managed by Maritime New Zealand led by Wildbase, Massey University, a trained and experienced oiled wildlife response team. Wildlife is protected under the New Zealand Marine Mammal Protection Act 1978, the Wildlife Act 1953, the Conservation Act 1987, and the Fisheries Act 1996.

What species are likely to be present in regions where models predict oil could spread is reasonably well understood; however, specific species abundance will vary with the time of year and other environmental factors. Based on the known species data, abundance, and distribution predicted during New Zealand’s austral summer (the time the drilling was to occur), it would be estimated that wildlife populations in these areas would consist of thousands of individual birds (Forest and Bird 2014) and potentially hundreds of pinnipeds (seals) and cetaceans (whales and dolphins; Stephenson et al. 2020). The predominant species at risk in the identified areas include yellow-eyed penguins (*Megadyptes* antipodes), pinnipeds, New Zealand sea lions (*Phocarctos hookeri*) and fur seals (*Arctocephalus forsteri*), and *Procellariiformes* spp., i.e., tubenose seabirds, Albatross (*Diomedea* and *Thalassarche* spp.), petrels (*Pelagodroma* and *Procellaria* spp.), prions (*Pachyptila* spp.), and shearwaters (*Puffinus* spp.) (Chilvers 2021).

Although the oil trajectory model did not predict that oil would reach shore if spilled (MetOcean 2019), several species in this area would track oil back to shore almost immediately after the oil release. These species would be foraging daily in the areas identified in the trajectory during summer because they will all be breeding and returning to shore daily to feed their young. The other significant wildlife consideration in a spill scenario with oil trajectory as predicted are the Procellariiformes, tubenose seabirds. There would be thousands of these birds foraging in this area during the drilling season, as the area is a known foraging hotspot (Forest and Bird 2014). Many of the Procellariiformes species are listed as endangered species and would be high-priority response species (Robertson et al. 2021).

The wildlife response operations for this New Zealand drilling site would be undertaken by trained personnel and would include on-land and at-sea reconnaissance, with part of the monitoring including the identification of possible at-sea hazing (the use of active or passive physical, visual or audible techniques to keep wildlife out or move unoiled wildlife out of an area which has been oiled or will soon be oiled), and capture or recovery areas. Search and recovery would be needed at the on-shore colonies of all species identified as foraging in the area oil has been seen, i.e., sea lions, fur seals, and albatross. The high number of animals possibly affected and the likely at-sea distribution of the oil means the reconnaissance, hazing, recovery, and transport operations for this response would be undertaken by multiple types of transport including vessels, planes, helicopters, and foot and road transport for on-land searching for breeding colonies.

Specific response operations would include the following:

1. Transport for reconnaissance, hazing, recovery, and release—would be undertaken from the air, and land at known breeding sites. At sea, operations would be required and would be dependent on vessel availability and environmental conditions. There are planes, helicopters, and vessels identified in regional and national New Zealand plans that would be utilized. Transport around the shore areas where oil-affected wildlife are likely to return to land or be washed up would be a combination of foot, vehicle (car and quadbike), and vessels.
2. Field stabilization sites would be needed at known seal and penguin colonies if these species track oil back from sea foraging. Given the area the oil could cover, debilitated and/or dead wildlife could wash up over more than 500 km of the South Island’s east coast including difficult-to-reach places. Reconnaissance and monitoring of the coastline will determine where field stabilization sites would be established. If at-sea search and recovery is established, field stabilization would need to be established on the vessel and at the most likely landing point for the vessel operations.
3. An oiled wildlife facility would be established at the closest large city to the oil release (Dunedin, Fig. 1) but depending on the extent of oil and the number and locations of oiled wildlife, a facility may also need to be established further north of the drilling site (Christchurch, Fig. 1). If seal species, particularly the endangered New Zealand sea lions are impacted, extreme care is required if responded to, to ensure they are not exposed to dogs to reduce any canine disease transfer. Therefore, a purpose-built facility or in situ cleaning/rehabilitation would be recommended.

## Planning for offshore oiled wildlife response in Brazil

**Fig. 2:**
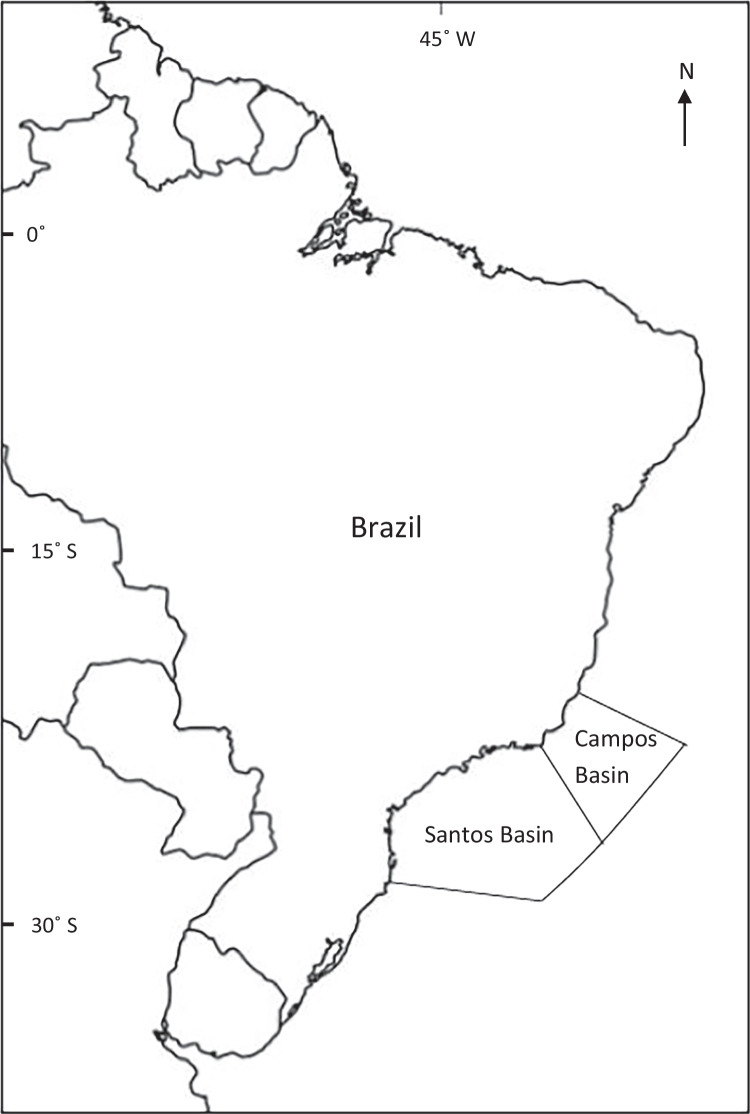
Location of Santos and Campos Basins, Brazil

The most significant oil fields in Brazil are in the Santos and Campos Basins, predominately over 250 km from shore near Rio de Janeiro, Brazil (Fig. 2). Oil was first searched for and found in both Basins in the early 1970s with production following later in the decade. Santos Basin contains one of the most productive pre-salt (oil reserve trapped below a thick layer of salt) areas for petroleum in the world. In 2017, the Campos Basin had a total of 863 offshore drilled wells, 537 of which produced oil, while the Santos Basin had 114 offshore drilled wells and 55 in production (Zacharias and Fornaro 2020). There have been several offshore oil spills reported from these basins with the latest being the unknown South Atlantic oil spill in 2019 (Lira et al. 2021) which was first identified on the Brazilian coast on the 30^th^ of August 2019. This spill had significant effects on wildlife and shoreline ecosystems with oil reaching 4334 km of coastline along the States of Sao Paulo and Rio de Janeiro.

The Brazilian Federal Law 9966 (2000) was enacted to regulate the prevention, control, and monitoring of the pollution caused by the release of substances into water and is overseen by the National Environment Council (CONAMA) Resolution 293 on December 12, 2001 (Ferreira et al. 2003). This resolution defines, among other things, the Individual Emergency Plan, which includes the identification of risks of potential sources of spills, discussion of accidental hypotheses, vulnerability analysis, and definition of potentially affected areas. There have been several alterations to the legislation with CONAMA Resolution 398 (2008) which relates specifically to oiled wildlife response which determines content for companies’ contingency plans and mandates: “Each plan should detail measures to be taken to rescue and protect the wildlife affected in case of an oil spill including a signed agreement with responders.” There is also a National Contingency Plan for Oiled Wildlife Response–IBAMA, 2016 which includes IBAMA’s actions, best practice guide, and species vulnerable to oil spills (www.marem-br.com.br). Environmental mapping for response to Emergency at Sea (MAREM) describes the vulnerability of the Brazilian coast to oil spills including ecologically important areas such as Abrolhos Bank and Trindade Archipelago and their fauna. These areas lay in the path of potential oil spills from the Santos and Campos Basins.

Based on the known species distribution and abundance in the areas likely affected by spills from either the Santos or Campos Basins, there would be marine sea turtles, marine mammals, and thousands of individual birds from many species impacted. Species at risk would include offshore species such as *Procellariiformes* spp., i.e., tubenose seabirds, Albatross (*Diomedea* and *Thalassarche* spp.), petrels (*Pelagodroma* and *Procellaria* spp.), prions (*Pachyptila* spp.), and shearwaters (*Puffinus* spp.) and nearshore species including teal (*Anatidae* spp.), Sandpipers (S*colopacidae* spp.), and Heron species (*Andeidae* spp.).

**Fig. 3:**
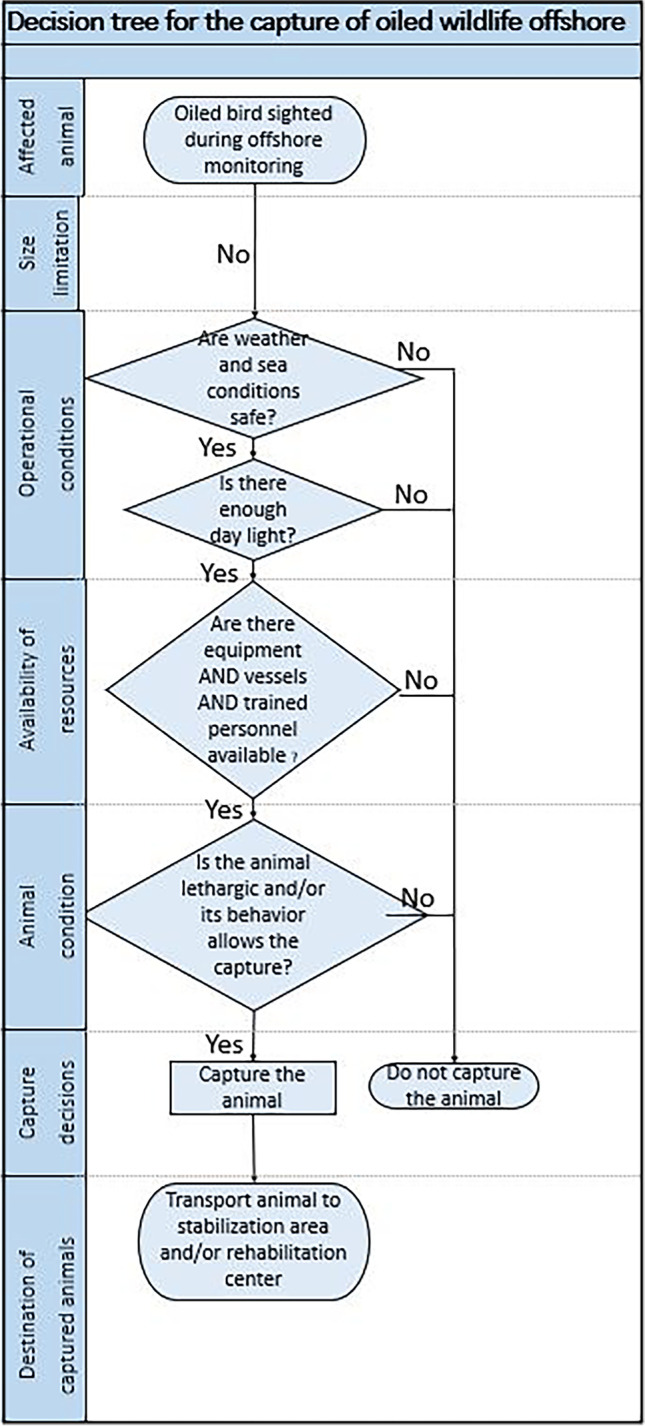
Decision tree for offshore capture of avian wildlife (sea birds). Developed by Aiuká, Brazil and Oiled Wildlife Care Network (OWCN) University of California Davis

The wildlife response operations in Brazil would be undertaken by trained personnel and would largely be on-water, offshore captures, and operations, as included in the wildlife protection plans given the distance offshore the oil extraction occurs. Examples of decision trees for the capture of wildlife at sea developed by Aiuká, Brazil, and the University of California, Davis, USA, are given in Fig. 3 and discussed further in the special considerations and procedures for offshore oiled wildlife response section below. On-water or air reconnaissance and monitoring would occur as well as at-sea hazing, capture, and recovery.

Specific response operations would include the following:

1. Transport for reconnaissance, hazing, recovery, and release will be undertaken from the air and at sea. Specific vessel types are identified in industry plans (Pers. Comm. Valeria Ruoppolo, Aiuká).
2. Field stabilization sites will be set up at sea on vessels and in areas on islands and the coastline where debilitated and/or dead wildlife could wash up. Reconnaissance and monitoring of all coastlines will determine where field stabilization sites will be needed. If at-sea search and recovery is established, field stabilization would need to be established on the vessel.
3. Affected wildlife will be returned to land (> 250 km) where Aiuká’s established oiled wildlife facilities are located in Praia Grande–Säo Paulo, and Rio das Ostras, Rio de Janeiro.

## Special considerations and procedures for offshore oiled wildlife response

For any offshore spill, the sooner wildlife teams are mobilized the better outcomes for the wildlife. At sea floating vegetation, remote islands, and other at-sea refuges such as drilling rigs and platforms will concentrate wildlife and are places where searches should start, along with known breeding areas onshore, where oiled wildlife may return to.

Procedures for capturing wildlife at sea have been previously determined by Aiuká, Brazil and Oiled Wildlife Care Network (OWCN) the University of California Davis, two of the world’s most experienced oiled wildlife response organizations. Aiuká and OWCN undertook a workshop “Offshore Wildlife Field Operations” on the feasibility, safety, and strategies needed for offshore hazing/deterrence, capture, and stabilization of oiled wildlife. An outcome of this workshop was the development of decision trees for the offshore capture of sea turtles, marine mammals, and seabirds (Fig. 3). Decision-making elements for the at-sea capture procedure included: animal size, operational conditions (such as meteorological and oceanographic conditions), resource availability, animal condition, and destination (Santos et al. 2018). This is the sort of pre-spill planning that is needed for a region or country to determine how it will run an oiled wildlife response. On-water capture of oiled wildlife has been performed previously during the Selendang Ayu and Macondo spills (Anonymous 2011). Pro-active capture like that undertaken at Selendang Ayu and Macondo increases wildlife survivability and release rates as animals that are captured sooner are likely to be in better condition (i.e., not suffering from dehydration, starvation, or thermoregulation difficulties such as hypothermia).

### Specific Taxa consideration Procellariiformes: tubenose birds

Birds are the taxa of wildlife most affected by oil spills and for the Southern Hemisphere, the Oceans are critical habitats for significant numbers of *Procellariiformes*, oceanic birds. Many of these species are endangered or threatened (https://www.iucnredlist.org) and therefore are considered high-priority species during an oiled wildlife response. This is not only because they forage in all southern oceans of the world but also for the Albatross and larger petrel and shearwater species; they are large enough that they could be light to moderately oiled and survive for days at sea, although not able to fly or feed. Therefore, from animal welfare and survival point of view, where possible, these species need to be assessed at sea and be considered for capture for rehabilitation (Fig. 3).

During an offshore oiled wildlife response, daily monitoring for wildlife is needed if possible, and depending on weather and access to equipment and personnel availability, at-sea capture possibilities should be regularly assessed to determine if can be undertaken. At-sea captures of *Procellariiformes* have been undertaken previously using floating mist nets, cast nets, net guns, and hoop nets (Bugoni et al. 2008; Ronconi et al. 2010; Trull et al. 2018), usually with the help of chumming or encouraging with food. Capture would be attempted from small boats which are likely to be tenders of larger, potentially live-aboard vessels that would also operate as an observation and monitoring platform, potential hazing or deterrence platform, retrieval of oiled carcasses platform, as well as capture and field stabilization vessel.

## Considerations for offshore capture Procellariiformes

Criteria for conducting offshore capture of oiled wildlife will need to be considered for each country or region depending on legislation, equipment, capability, and personnel experience (Fig. 3, developed by Aiuká, Brazil and Oiled Wildlife Care Network (OWCN) University of California Davis).

- Affected animal: in most situations, all avian species affected by oil can be captured.
- Wildlife size limitation: there is unlikely to be size/weight limitations for birds as even the heaviest sea bird in the world is less than 15 kg. Understanding size limitations ensures operational cut-offs are known, resource/equipment availability is understood, and teams are trained to these limitations.
- Operational conditions: the offshore capture can only be considered viable if there are enough daylight hours, and all the weather (wind, ≤ Beaufort 3) and oceanographic conditions (wave height and swell) allow safe operation.
- Availability of resources: the capture may only be carried out if trained personnel, all necessary equipment, and appropriate vessels are available. It is essential to obtain all three simultaneous resources, and in the absence of one of them, there will be no offshore capture. Pre-identified personnel and resources can mobilize quickly. Ideally, a specific or purpose-built vessel available that has ready access to waterline for retrieval of wildlife, areas for containment and protection of wildlife, wildlife examination, and stabilization areas, oiled waste management (containment) areas, as well as the ability to transport wildlife back to land once a day (or ability to transfer to helicopter or another fast vessel).
- Animal condition: in general, all oiled birds could be captured.
- Destination of captured animals: all bird species will need to be stabilized and then sent to a wildlife rehabilitation center for decontamination, rehabilitation, and waterproofing before release. As most *Procellariiformes* are capable and used to traveling enormous distances during their lives and often during single foraging trips, individuals could be released from the shore near the rehabilitation center, which is hopefully also presumably outside the area of the oil spill. The same would need to occur for any other wildlife species considered for capture except sea turtles in good nutritional/hydration condition as assuming the facilities existed onboard or on a nearby vessel, these animals could be cleaned in situ and released back into clean water.
- Euthanasia: consideration of euthanasia should be taken on a case-by-case basis based on animal ethics/welfare, cultural, and legal considerations for each area/country.
- Trained and experienced personnel: personnel need to be identified, exercised, and drilled in equipment use, on-water capture, and all necessary health and safety training which may include for example helicopter underwater escape training (HUET), or Basic Safety Training for Platforms/ Basic Offshore Safety Induction and Emergency Training (BOSIET).

## Conclusion and recommendations

This paper concentrated on offshore oil spill response; however, the same recommendations and preparedness for oiled wildlife response are needed for all situations, regions, and countries (Ruoppolo et al. 2013; Chilvers 2021). Oil drilling is increasingly happening in remote offshore locations (Bergstrom and Selkirk 2007; Crawford et al. 2017; Rodríguez et al. 2019) and with increased drilling comes increased risks of accidents and oil spills. These increases need to be accompanied by a similar increase in awareness, preparedness, and planning for both an oil spill and oiled wildlife response. The fundamental principle of wildlife response is to prevent wildlife from getting oiled in the first place. However, if an oil spill does occur, the goal is to minimize wildlife contact with oil, and last when wildlife is oiled to capture as quickly, safely, and effectively as possible, clean, rehabilitate, and release into a clean environment. This means recovery of oil and wildlife on the water at sea are the most effective tools for the overall goals of oiled wildlife response in offshore spills, when practical and appropriate dispersant use could also be considered. However, care needs to be taken with dispersant use around wildlife, because dispersants on birds’ feathers can be as debilitating and lead to death from hypothermia and drowning in the same way as oil by disrupting the birds feather structure (Whitmer et al. 2018). On-water recovery is what an offshore response should plan for to maximize the protection of the coastline and increase the survival of the wildlife affected. Planning for offshore response helps oil companies and regulatory agencies to understand the challenges of this kind of response and enhances the importance of specific safety and preparedness (personnel and equipment) for all response types. Exercising plans is equally important to understand feasibility, and readiness and to allow continuous improvement of plans. Overall, there are obvious technical and logistical challenges for responding in offshore areas. However, the benefits of having developed plans, preparation of specialized equipment, and readiness of skilled trained response personnel encourage the prevention and minimization of impacts from offshore oil spills.

## Data Availability

This is not applicable.
